# The complete chloroplast genome sequence of *Zingiber teres* S. Q. Tong & Y. M. Xia (Zingiberaceae)

**DOI:** 10.1080/23802359.2023.2226256

**Published:** 2023-06-25

**Authors:** Xiao Wang, Shuming Tian, Hao Wang, Lin Yang, Xiaoling Zou, Xavier-ravi Baskaran, Qiang Li, Haitao Xing, Hong-Lei Li

**Affiliations:** a College of Landscape Architecture and Life Science, Chongqing University of Arts and Sciences, Chongqing, China; b Savari Research Foundation/Savari Naturals, Tirunelveli, Tamil Nadu, India

**Keywords:** *Zingiber teres*, chloroplast genome, phylogeny, species identification

## Abstract

Here, the complete chloroplast genome sequence of *Zingiber teres* is described using MGI paired-end sequencing. The genome is 163,428 bp in length and contains a small single-copy region (SSC) of 15,782 bp, a large single-copy region (LSC) of 88,142 bp, and two inverted repeat (IR) regions of 29,752 bp. The overall GC content is 36.1%, and the GC content of the IR regions is 41.1%, which is higher than that of both the LSC region (33.8%) and SSC region (29.5%). The genome of *Z. teres* contains 133 complete genes, including 88 protein-coding genes (79 protein-coding gene species), 38 tRNA genes (28 tRNA species), and 8 rRNA genes (four rRNA species). Maximum likelihood phylogenetic analysis yielded a well-resolved tree of the genus *Zingiber*, and *Z. teres* and *Zingiber mioga* were sister species in this tree. The development of DNA barcodes could aid the identification of *Zingiber* species.

## Introduction

*Zingiber teres* S. Q. Tong & Y. M. Xia (Tong and Xia 1987) is a member of the genus *Zingiber* in the family Zingiberaceae. *Z. teres* occur at high elevations in hill evergreen forests in southern China and northern and northeastern Thailand (Tong and Xia 1987; Triboun et al. 2007). The height of the slender stem of *Z. teres* ranges from 60 to 100 cm; *Z. teres* can be distinguished from other *Zingiber* species by the length of the ligule (2–4 mm) and petiole (ca. 2 mm) (Wu and Larsen 2000). The rhizome of *Z. teres* is cylindrical and has an aromatic odor similar to that of other ginger species. The elliptic inflorescences arise from rhizomes with peduncles embedded in the ground. *Z. teres* has several features that would potentially make it an excellent ornamental plant. *Zingiber* plants are difficult to identify because of their short and seasonal flowering cycles, the high degree of phenotypic plasticity in many of their traits, and their high morphological similarity (Theerakulpisut et al. 2012; Basak 2014). DNA barcoding is an effective technique for the identification of species (Hebert et al. 2003; Chen et al. 2010). Divergent hotspots that could be used for the identification of species in the subfamily Zingiberoideae have been previously identified (Li et al. 2021a, 2021b). The chloroplast genome of *Z. teres* has not yet been described. Here, we sequenced and assembled the whole chloroplast genome of *Z. teres*. This chloroplast genome will enhance our understanding of the population genetics of *Z. teres* as well as the evolutionary history of the genus *Zingiber*.

## Materials

**Figure 1.**
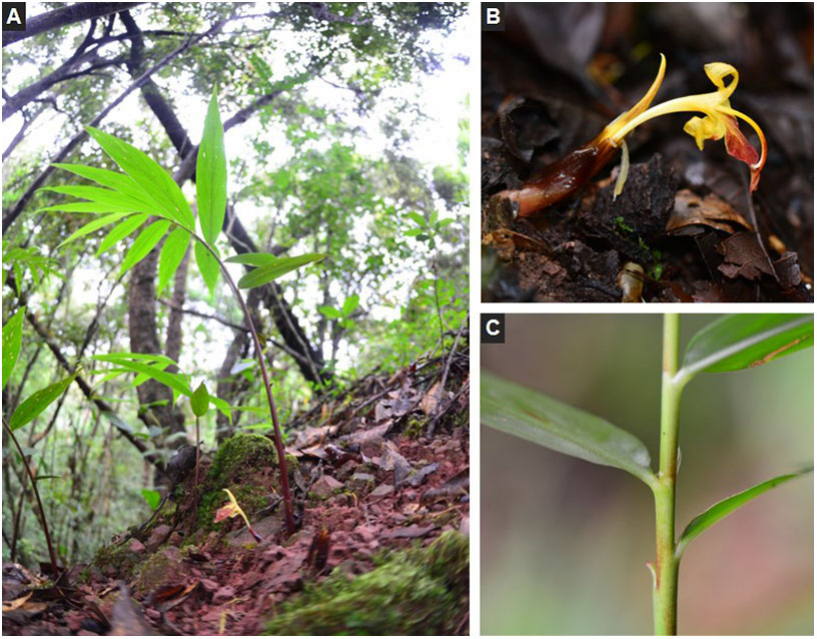
The morphological characteristics of *Z. teres*. A, the photo of whole plant; B, flower; C, stem (photos taken by Renbin Zhu at Laiyanghe Park in Puer, Yunnan).

The *Z. teres* (Figure 1) material sequenced in this study was collected from Laiyanghe Park in Puer, Yunnan Province, China (22.58°N, 101.06°E). This species is common in southwest Yunnan and can be collected without permits. Fresh and healthy leaves were dried with silica gel and preserved for DNA extraction. A specimen was deposited at the Herbarium of Chongqing University of Arts and Sciences (www.cqwu.net, Huamin Liu, liuhuanin@126.com) under the voucher number Li_Z032.

## Methods

Total genomic DNA was extracted from silica gel-dried leaves using the modified sucrose gradient centrifugation method (Li et al. 2012). A NanoDrop 2000 micro spectrometer (Wilmington, DE, USA) was used to evaluate the concentration of isolated genomic DNA; the genomic DNA was then stored in a −80 °C refrigerator at the Key Laboratory of the College of Landscape Architecture and Life Science. The MGIEasy PCR-Free DNA Library Prep kit (MGI-TECH) was used to generate the paired-end DNA library with an insert size of 350 bp; the MGI DNBSEQ-T7 platform (MGI-TECH, Shenzhen, China) was then used to sequence the library. A total of 5.5 G raw data were produced. Clean data were obtained by removing the adaptor and low-quality sequences. The software NOVOPlasty3.5 (Dierckxsens et al. 2017) was used to *de novo* assemble the whole chloroplast genome; the k-mer length was 39 bp, and a fragment of the *rbcL* gene from *Zingiber officinale* was used as the seed sequence. Geneious v11.0.4 was used to annotate and manually correct the chloroplast genome using the *Z. officinale* (MW602894) genome as the reference genome sequence. The circular chloroplast genome maps of *Z. teres* were built using the OGDRAW v1.3.1 program with default settings.

A phylogeny of the genus *Zingiber* was built using the chloroplast genome sequence of *Z. teres*, along with those of seven other species, which were obtained from GenBank: *Zingiber recurvatum*, *Zingiber mioga*, *Z. officinale*, *Zingiber montanum*, *Zingiber corallinum*, *Zingiber spectabile*, and *Zingiber zerumbet. Kaempferia gaalanga* was used as the outgroup in the phylogenetic analysis. MAFFT was used to align the complete plastome sequences with a gap opening penalty of 2–2.5 and a gap extension penalty of 0. Phylogenetic trees based on these aligned sequences were built using the maximum likelihood (ML) method under the GTRGAMMA model; branch support was evaluated using 1000 bootstrap replicates. The software Geneious R11 was used to conduct the above analyses.

## Results

**Figure 2.**
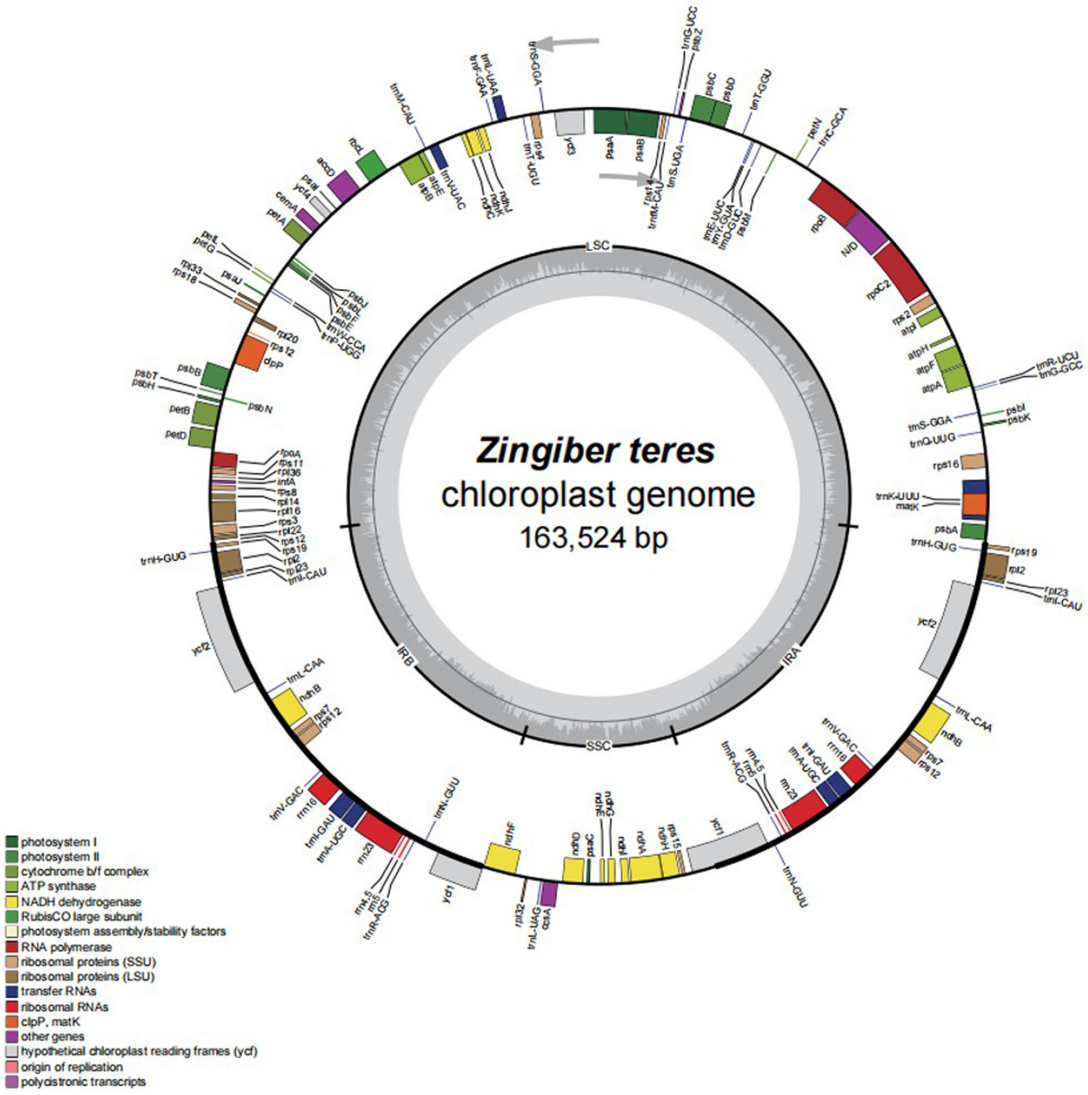
Map of the whole *Z. teres* chloroplast genome. Genes marked outside the circle are transcribed counterclockwise, and genes marked inside the circle are transcribed clockwise. The gray arrow indicates gene orientation. The tRNA genes are indicated by the one-letter code of amino acids with anticodons. LSC, large single-copy region; IR, inverted repeat; SSC, small single-copy region. Genes belonging to different functional groups are shaded differently. The innermost first ring in black indicates the chloroplast genome size of *Z. teres*. The innermost darker gray indicates the GC content, and the lighter gray indicates the at content.

The chloroplast genome of *Z. teres* was 163,428 bp (Figure 2, S1) and contained a small single-copy (SSC) region of 15,782 bp, a large single-copy (LSC) region of 88,142 bp, and two inverted repeat (IR) regions of 29,752 bp. The overall GC content was 36.1%; the GC content of the IR regions, LSC region, and SSC region was 41.1%, 33.8%, and 29.5%, respectively. The sequenced chloroplast genome contained 133 predicted functional genes, including 88 protein-coding genes (79 protein-coding gene species), 38 tRNA genes (28 tRNA species), and 8 rRNA genes (four rRNA species). (Figure 2). In total, 10 cis-splicing genes including rps16, atpF, rpoC1, ycf3, clpP, petB, petD, rpl16, rpl2, ndhB and ndhA (Figure S2A), and one trans-splicing genes rps12 (Figure S2B) were detected by CPGview (Liu et al. 2023) (http://www.1kmpg. cn/cpgview/).

**Figure 3.**
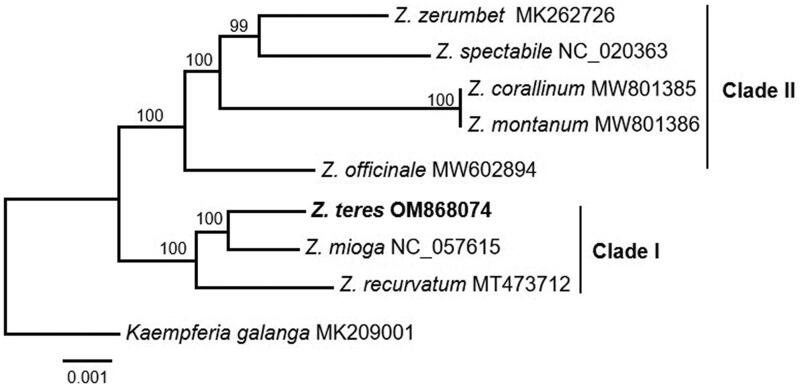
Maximum likelihood (ML) analysis of *Z. teres* and other related species based on the complete chloroplast genome sequence. The following sequences were used: *Z. teres* (OM868074), *Z. recurvatum* (MT473712; Li et al. 2021a), *Z. mioga* (NC_057615; Jiang et al. 2023), *Z. officinale* (MW602894; Jiang et al. 2023), *Z. montanum* (MW801386; Tian et al. 2023), *Z. corallinum* (MW801385; Tian et al. 2023), *Z. spectabile* (NC_020363; Tian et al. 2023), *Z. zerumbet* (MK262726; Li et al. 2021a), and *Kaempferia gaalanga* (MK209001; Li et al. 2019a).

We generated a well-resolved phylogeny of *Zingiber* using a chloroplast genome dataset (Figure 3). The bootstrap support values of all the branches were greater than 99%. Thus, the support values are not provided in the text below. *Zingiber* was divided into two main clades. Clade I comprising *Z. recurvatum* was sister to *Z. mioga* + *Z. teres*; clade II including *Z. officinale* was sister to (*Z. montanum* + *Z. corallinum*) + (*Z. spectabile* + *Z. zerumbet*).

## Discussion and conclusion

The chloroplast genome of *Z. teres* was reported for the first time in this study. The size (163,428 bp), GC content (36.1%), gene composition, quadripartite structure, protein-coding genes, tRNAs, and rRNAs of the *Z. teres* chloroplast genome were similar to those of other Zingiberoideae chloroplast genomes (Cui et al. 2019; Li et al. 2019a, 2019b). Many plants in families such as Malvaceae, Araceae, and Rhamnaceae have chloroplast genomes with the same gene content and gene order (Waseem et al. 2020; Henriquez et al. 2021; Mehmood et al. 2021a, 2021b). Genome variations such as plastome rearrangement, gene loss, intron loss, and gene duplication are common in plants (Lin et al. 2015; Daniell et al. 2016, Lee et al. 2020; Li et al. 2021a). The loss of both *trnS-GGA* and *trnT-GGU* has been reported in the chloroplast genome of *Globba schomburgkii*, which is in the family Zingiberoideae. The *lhbA* gene has been lost in both *Hedychium coccineum* and *Hedychium neocarneum* (Li et al. 2021a). However, the chloroplast genomes of *Z. teres* were highly conserved. Hence, specific molecular mechanisms might be responsible for the conservation of the *Z. teres* plastomes, including the rarity of plastid fusion, uniparental inheritance, and the presence of an active repair mechanism, as has been reported in previous studies (Wicke et al. 2011; Mehmood et al. 2021b).

Our phylogeny of *Zingiber* was well resolved, and all the branches were highly supported. The relationships among *Zingiber* species were similar to those inferred in previous studies (Theerakulpisut et al. 2012; Li et al. 2021a). *Z. recurvatum* was grouped with *Z. teres* + *Z. mioga* and sister to clade II. This result is consistent with a previously published molecular phylogeny in which *Z. recurvatum* was sister to the other *Zingiber* species (Li et al. 2021a; equivalent to clade II in this study). Our results showed that *Z. zerumbet* was sister to *Z. spectabile* with strong support. Similar relationships have also been observed in previous studies (Theerakulpisut et al. 2012; Li et al. 2021a). In general, the support values of each branch within our phylogeny of *Zingiber* were higher than those of a previously published phylogeny based on nuclear genome data (Theerakulpisut et al. 2012; Li et al. 2021a). These results indicate that the chloroplast genome has a sufficient number of informative sites to resolve the phylogenetic relationships among *Zingiber* species. The development of DNA barcodes could aid the identification of *Zingiber* species.

## Supplementary Material

Supplemental Material

## Data Availability

The genome sequence data that support the findings of this study are openly available in GenBank of NCBI at (https://www.ncbi.nlm.nih.gov/nuccore/OM868074) under the accession no. OM868074. The associated BioProject, SRA, and Bio-Sample numbers are PRJNA827347, SRR18899755, and SAMN27609002 respectively.
