# Supplementary material for: The complete chloroplast genome sequence of Zingiber teres S. Q. Tong & Y. M. Xia (Zingiberaceae)
Source: Mitochondrial DNA B Resour. 2023 Jun 25;8(6):699–703. doi: 10.1080/23802359.2023.2226256 (PMC10294729; doi:10.1080/23802359.2023.2226256)
Supplement: Supplemental Material [file TMDN_A_2226256_SM4034.docx]

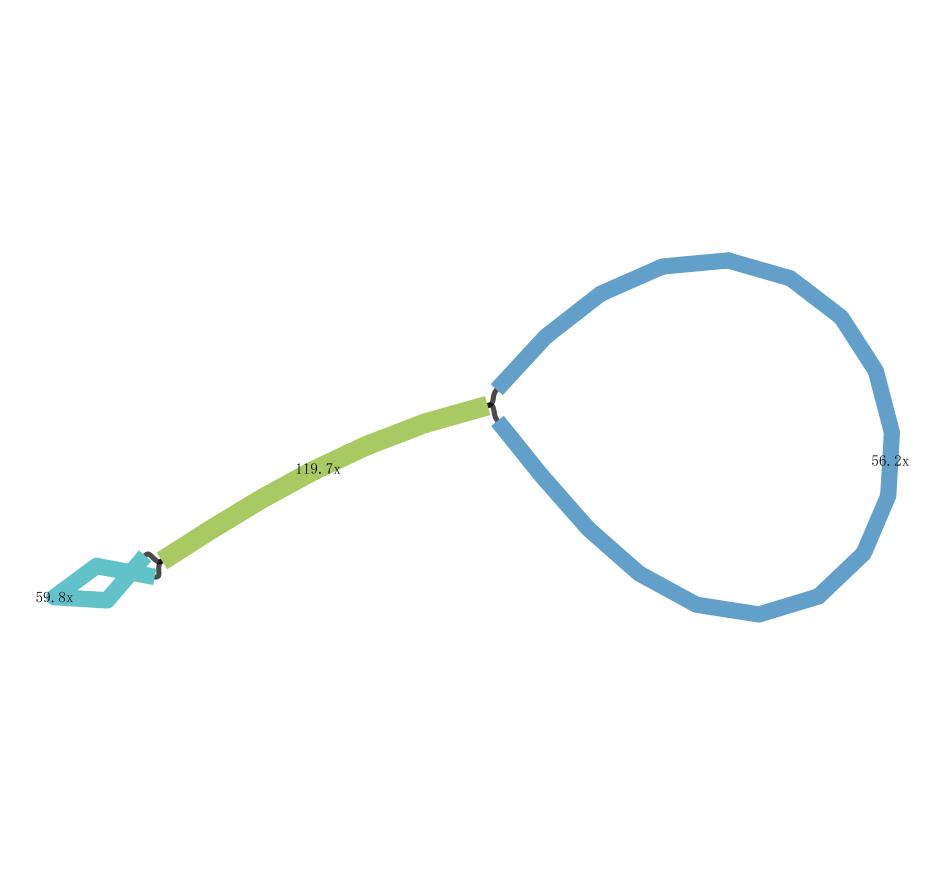


**Figure S1.** The assembly coverage plot for the *Zingiber teres* complete chloroplast genome by GetOrganelle.


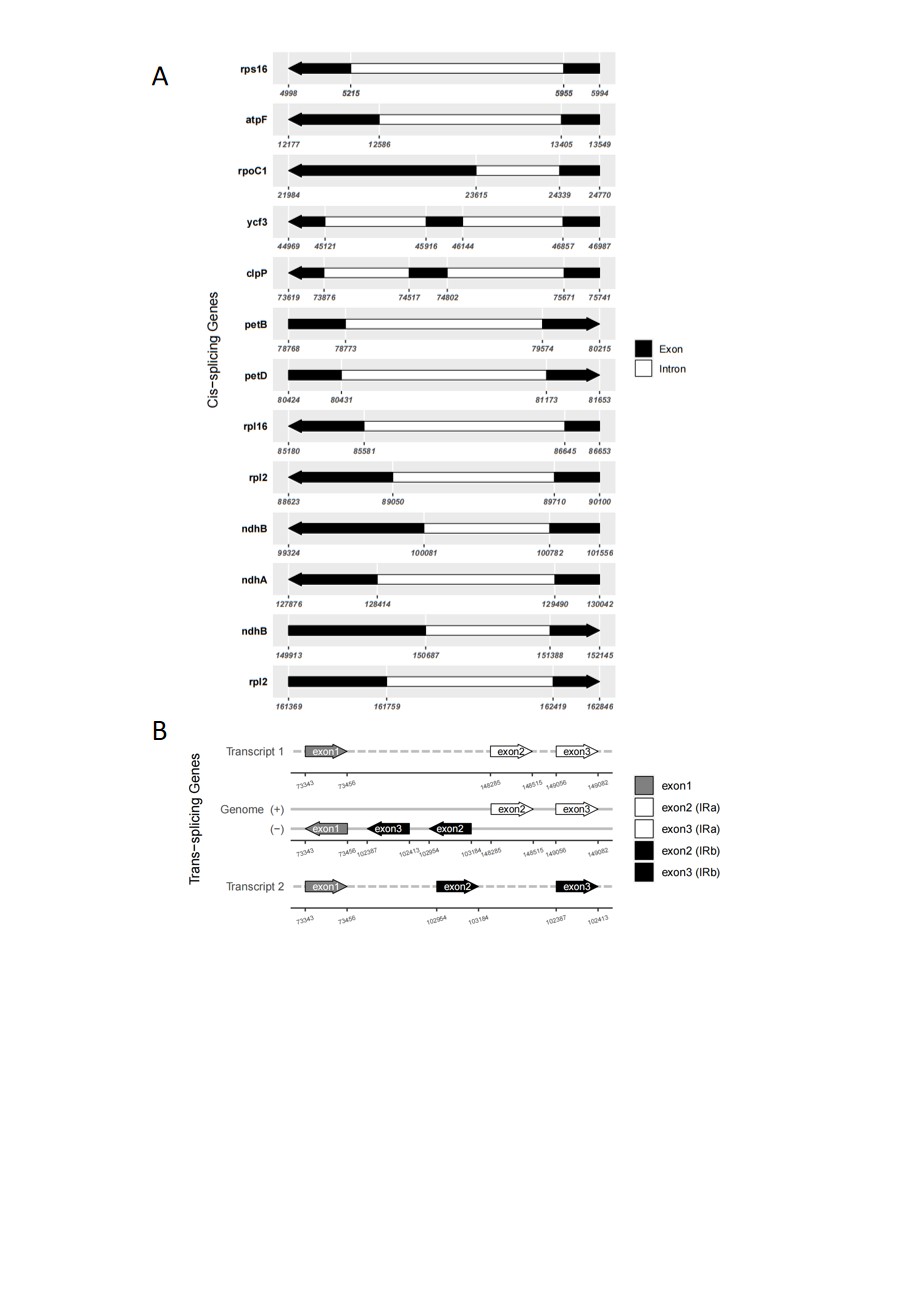


**Figure S2.** The structure of the 10 cis-splicinggenes (A) and one trans−splicing gene (rps12, B) annotated in Cp genome of *Zingiber teres*. The numbers in the figure represent the location in the Cp genome. The results were generated by CPGview.
